# Supplementary figures and images for: Leveraging expression from multiple tissues using sparse canonical correlation analysis and aggregate tests improves the power of transcriptome-wide association studies
Source: PLoS Genet. 2021 Apr 8;17(4):e1008973. doi: 10.1371/journal.pgen.1008973 (PMC8057593; doi:10.1371/journal.pgen.1008973)

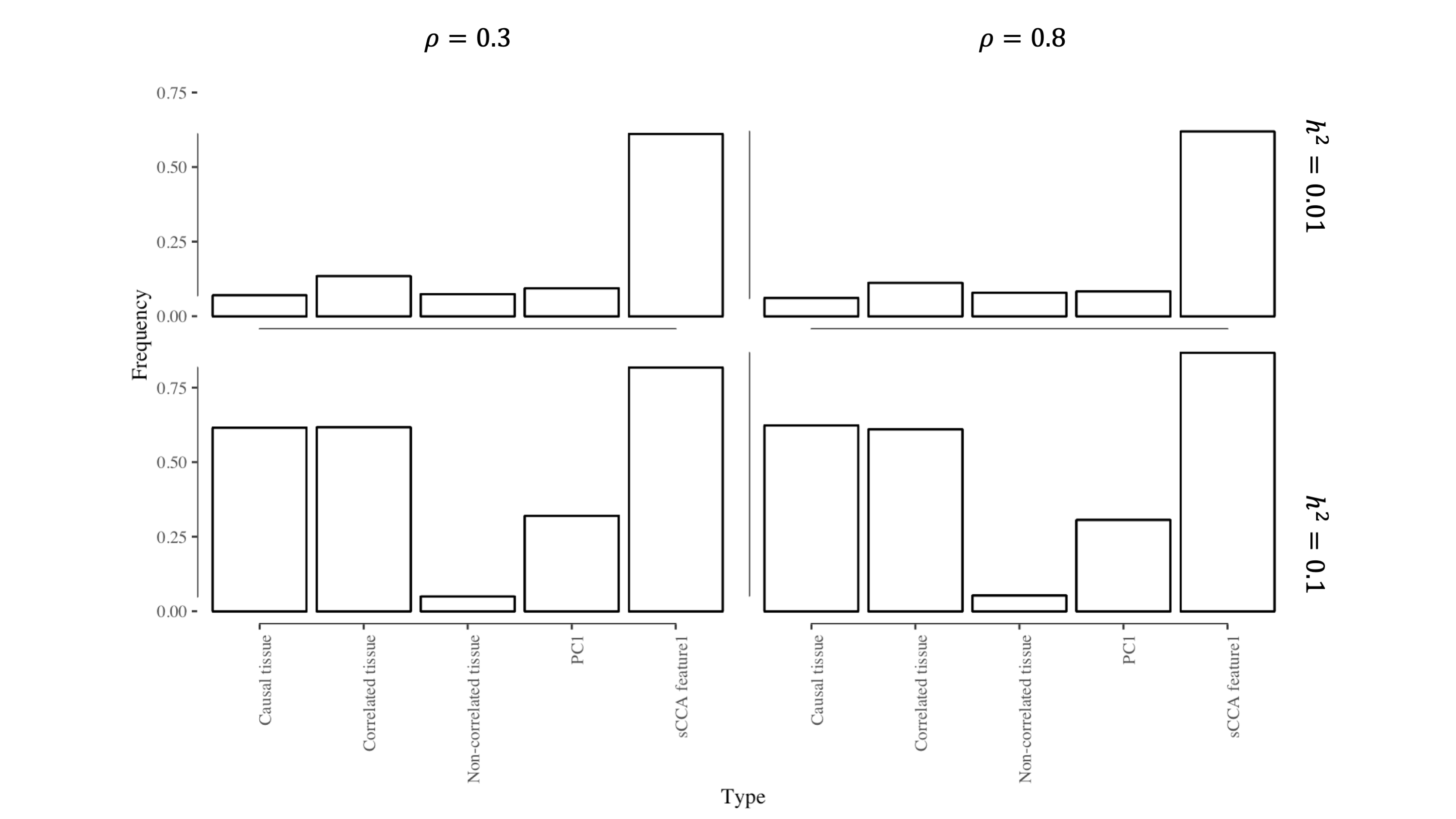

Supplement: S1 Fig — ρ denotes the strength of the genetic correlation between expression in the causal tissue and tissues where expression is also associated with cis germline variation ("correlated tissues"). "Non-correlated tissues" are tissues where local germline variation is not associated with gene expression. Here expression in all of the tissues is genetically correlated with the causal tissue, and the causal tissue is not observed (performance in the causal tissue is included as a reference). PC1 is the first principal component of cross-tissue gene expression; sCCA-feature1 is the linear combination of tissue expression values from the first pair of sCCA canonical variables. h2 denotes the proportion of expression variance in the causal tissue explained by cis genetic variation. (TIFF) [file pgen.1008973.s001.tiff]

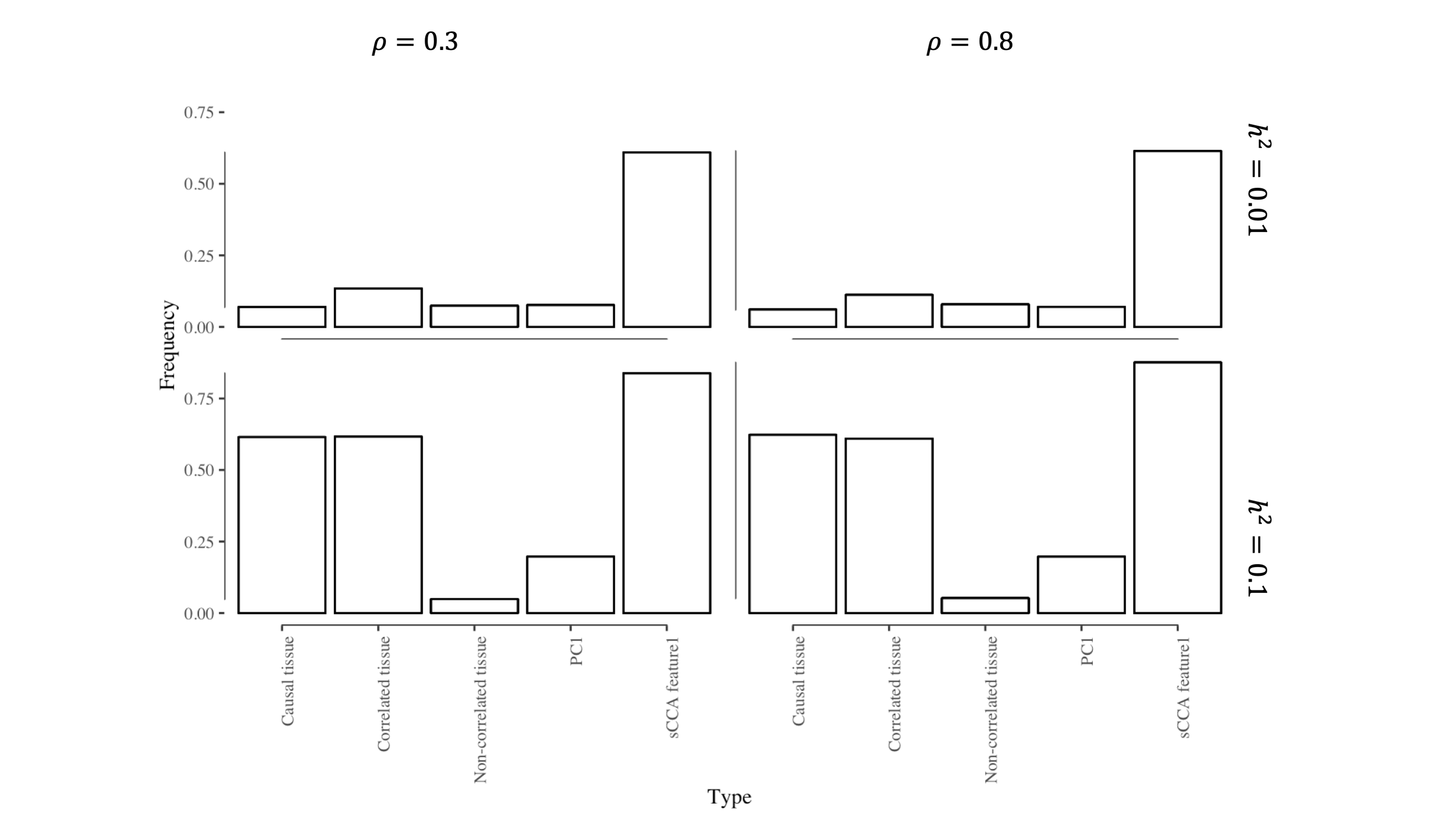

Supplement: S2 Fig — ρ denotes the strength of the genetic correlation between expression in the causal tissue and tissues where expression is also associated with cis germline variation ("correlated tissues"). "Non-correlated tissues" are tissues where local germline variation is not associated with gene expression. Here expression in half of the tissues is genetically correlated with the causal tissue, and the causal tissue is observed. PC1 is the first principal component of cross-tissue gene expression; sCCA-feature1 is the linear combination of tissue expression values from the first pair of sCCA canonical variables. h2 denotes the proportion of expression variance in the causal tissue explained by cis genetic variation. (TIFF) [file pgen.1008973.s002.tiff]

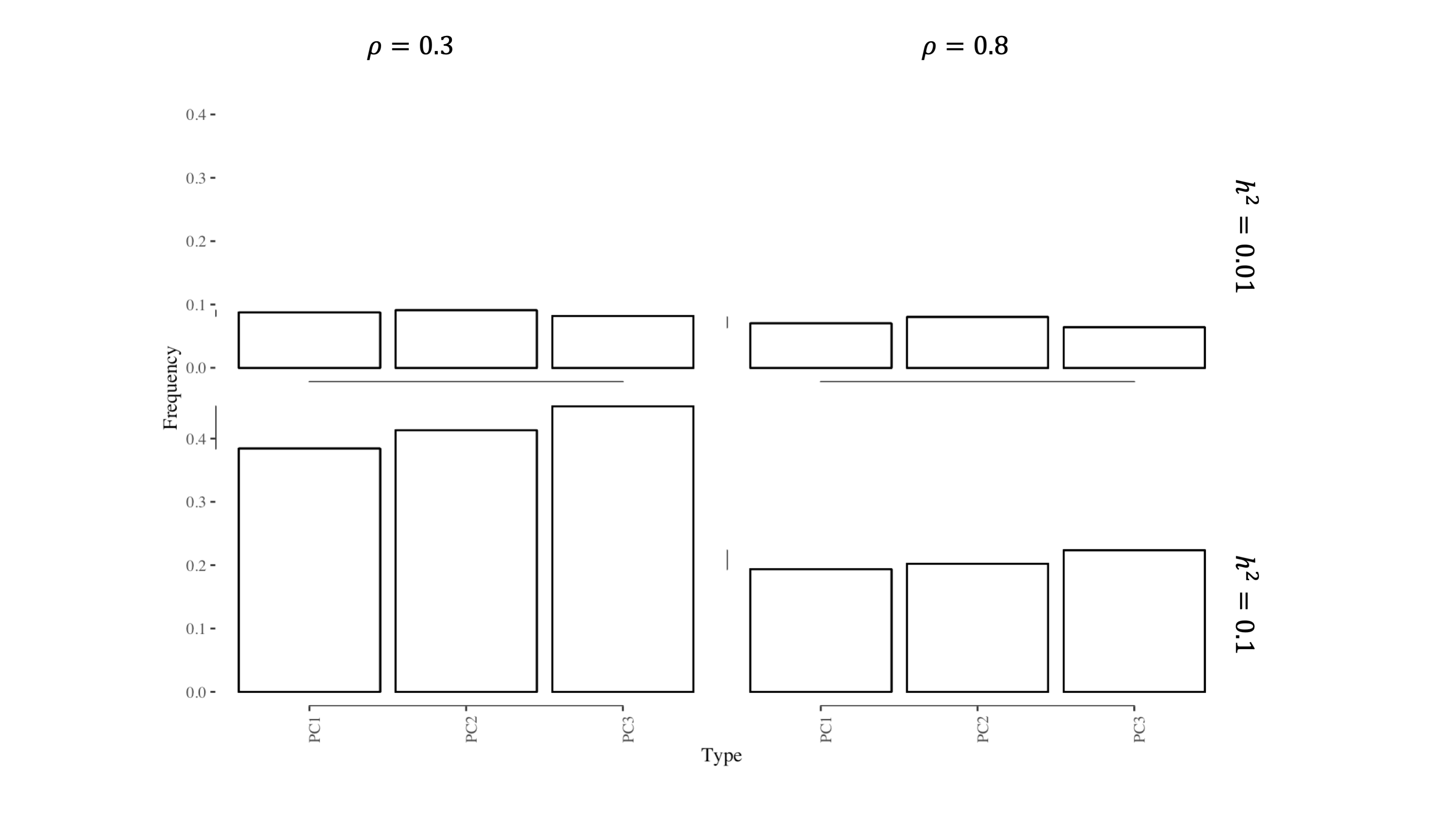

Supplement: S3 Fig — ρ denotes the strength of the genetic correlation between expression in the causal tissue and tissues where expression is also associated with cis germline variation. Half of the tissues are genetically correlated with the causal tissue, which is not observed. h2 denotes the proportion of expression variance in the causal tissue explained by cis genetic variation. (TIFF) [file pgen.1008973.s003.tiff]

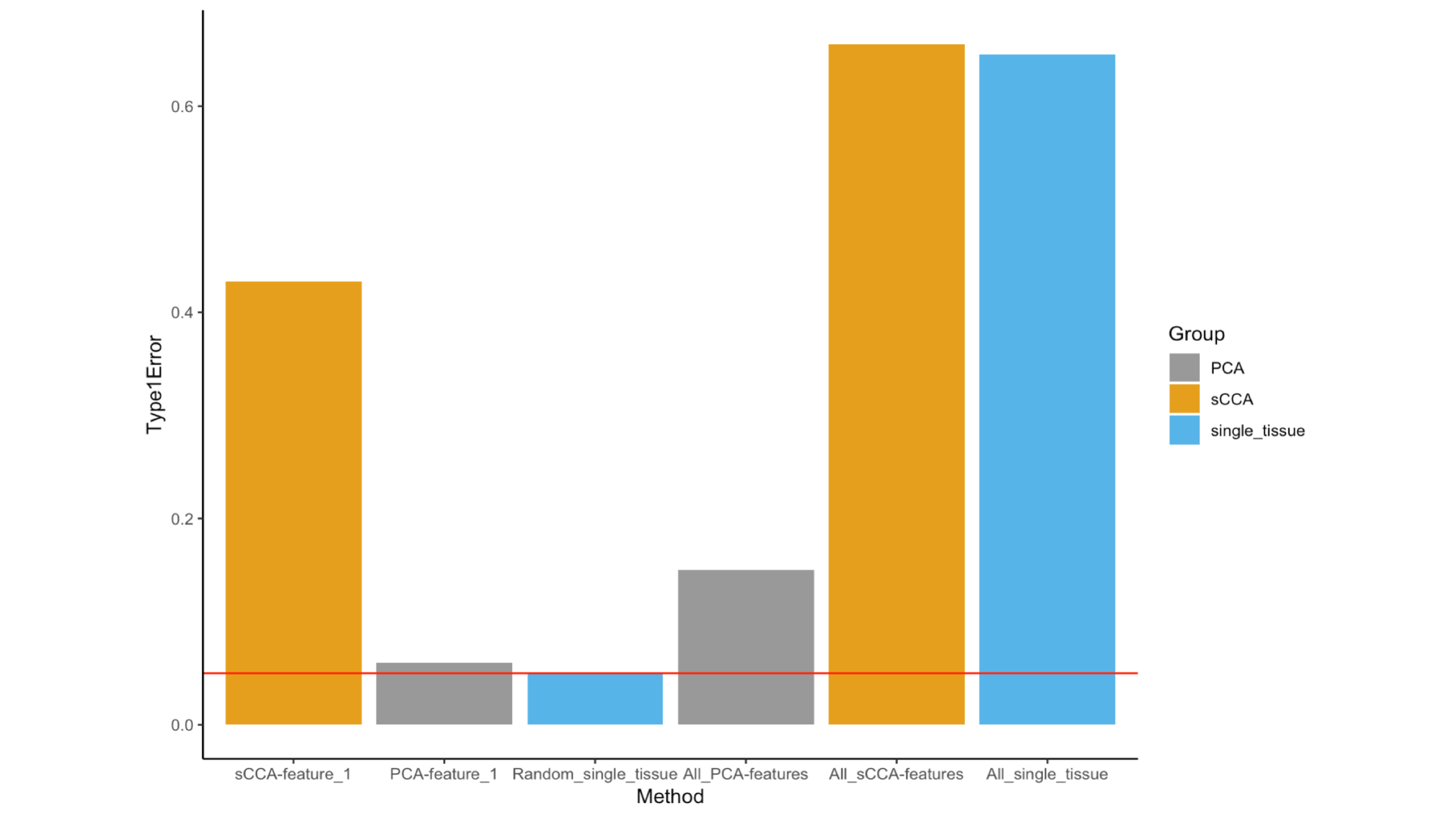

Supplement: S4 Fig — Proportion of simulations where local genetic variation was nominally statistically significantly associated with gene expression, in the scenario where no association was present. sCCA-Feature_1: testing only the leading sCCA expression feature at the α = 0.05 level; PCA-feature_1: testing only the lead cross-tissue expression principal component at the α = 0.05 level; All_PCA-features and All_sCCA-features: proportion of simulations where at least one of the top three PCA (resp. sCCA) features was significant at the α = 0.05 level; All_single_tissue: proportion of simulations where at least one of the 22 single-tissue tests was significant at the α = 0.05 level. (TIFF) [file pgen.1008973.s004.tiff]

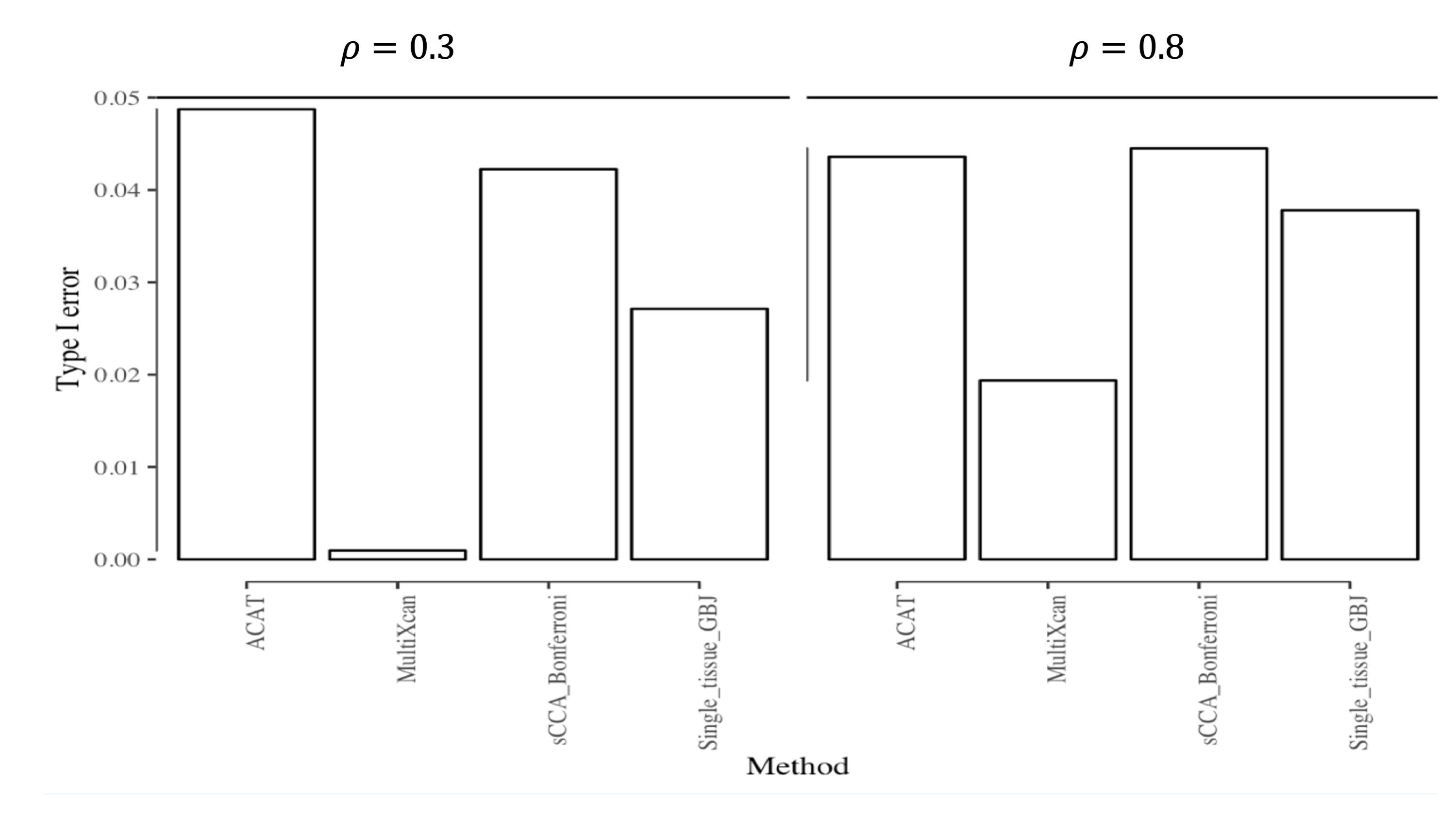

Supplement: S5 Fig — Proportion of significant results under the (gene expression not associated with phenotype) averaged over all scenarios. (TIFF) [file pgen.1008973.s005.tiff]

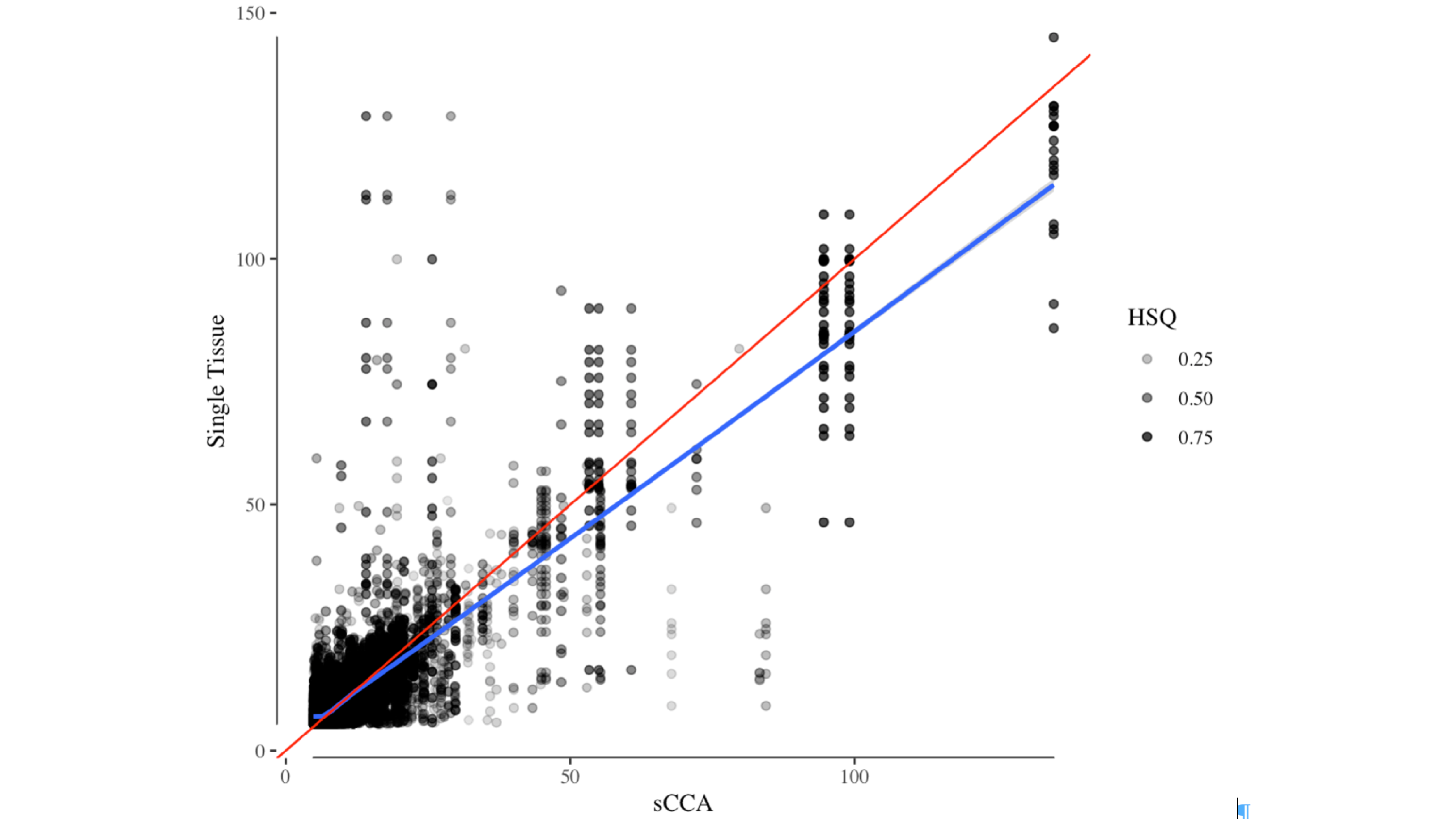

Supplement: S6 Fig — The TWAS test statistics using sCCA feature 1 and all single tissue weights from Fusion are plotted on the x-axis and y-axis respectively. The blue line is the fitted regression line and red line is y = x. (TIFF) [file pgen.1008973.s006.tiff]

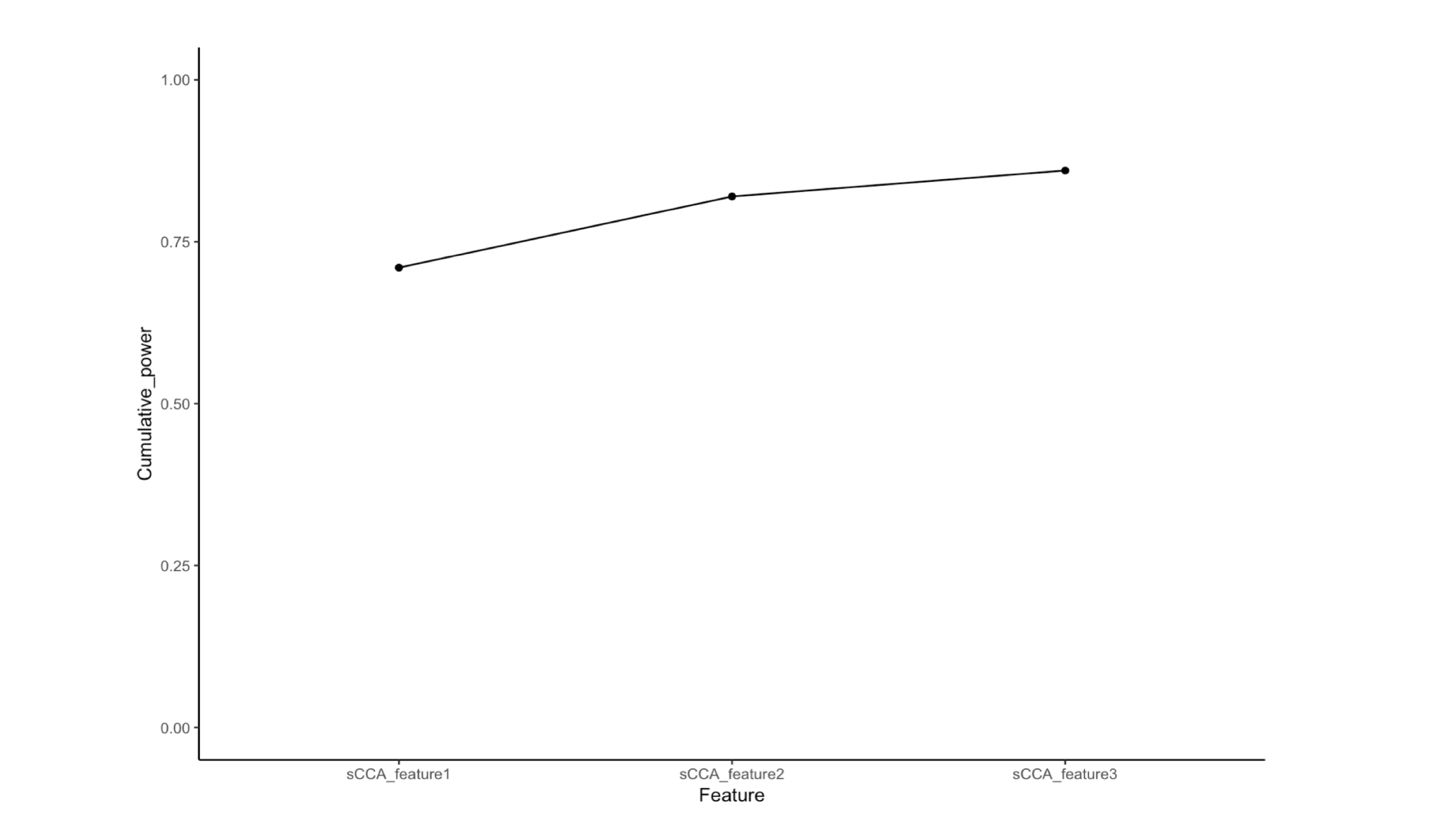

Supplement: S7 Fig — The Y axis indicate the cumulative power of detecting heritable genes when include only sCCA feature 1, sCCA feature 1 and 2, and sCCA feature 1 to 3, average over all scenarios. (TIFF) [file pgen.1008973.s007.tiff]
