# Supplementary material for: Leveraging expression from multiple tissues using sparse canonical correlation analysis and aggregate tests improves the power of transcriptome-wide association studies
Source: PLoS Genet. 2021 Apr 8;17(4):e1008973. doi: 10.1371/journal.pgen.1008973 (PMC8057593; doi:10.1371/journal.pgen.1008973)
Supplement: Attachment — Submitted filename: Comments to the Authors_1110.docx [file pgen.1008973.s014.docx]

We thank the reviewers for their detailed comments, which have improved the paper’s clarity and broadened its scope (e.g. the inclusion of a direct comparison to UTMOST). Detailed responses to reviewers’ comments follow.

**Reviewer #1**

The paper describes a generalization of the TWAS pipeline that uses data across multiple tissue. The generalization has the potential for increasing power of detecting associations. It seems to me that the main contribution of the paper is the use of multi-tissues gene expression features (weighted combination of gene expression measurements) in the TWAS pipeline. The idea of using multiple tissues is not new (see for example MultiXcan) but the proposed implementation here is novel - it relies on constructing better features using sparse canonical correlation. The performance of the method in simulations and real data is investigated. The Abstract describes a models increase in power when compared to other methods.

Is there software available for this methodology? How about a database of features? I am not sure how the community will benefit from this paper without them.

We plan to make all sCCA cross-tissue feature weights available on the TWAS HUB web interface ([www.twas-hub.org](http://www.twas-hub.org)). These cross-tissue weights can then be used in TWAS analyses using existing software originally designed for single-tissue weights (e.g. FUSION <http://gusevlab.org/projects/fusion/> or MetaXcan https://github.com/hakyimlab/MetaXcan).

For learning new cross-tissue features and conduting cross-tissue TWAS, we have provided a sample pipeline (<https://github.com/fenghelian/sCCA-ACAT_TWAS>). We used existing software to conduct sCCA analysis, to construct expression feature weights, and combine single- and cross-tissue tests (specifically: penalized matrix decomposition for sCCA [the CCA function in the Penalized Multivariate Analysis R package], FUSION <http://gusevlab.org/projects/fusion/#computing-your-own-functional-weights>, and ACAT <https://github.com/yaowuliu/ACAT>). We also have information on these software packages to the manuscript Line153-159.

The sCCA-TWAS methodology as described is insufficient. As the authors mention in the paper in the context of GTeX data - not all subjects have measurements for all tissues. This is an interesting methodological problem that is also essential for an efficient implementation - how to deal with missing gene expression data? There are challenges due to the non-random nature of missing data (for example, when one brain tissues is missing, it is very likely that most other brain tissues will not be available as well). The simple solution of using only the subset of subjects that have data for all tissues is clearly inefficient. The proposed solution (on page 14) is also unsatisfactory - the authors propose to use an off-the-shelf method for imputation, MICE, that has not been validated as an accurate method for gene expression imputation. The proposed method controls Type 1 error (it is trivial to see why) but loses a great amount of power compared to complete data (0.38 to 0.21). It is clear that the authors miss an opportunity to increase power by doing a careful evaluation of how to deal with missing expression data. This can be done in the context of sparse canonical correlation via structured equations or by using imputation methods that have been developed in the context of gene expression data.

The reviewer raises excellent points, which we have added to the discussion as an important area for future work (lines 415-417). We agree that our approach to missing data could likely be improved, but our primary focus here was to explore the utility of sCCA for constructing multi-tissue features. We show that sCCA outperforms PCA and single-tissue TWAS approaches--even when an ad hoc, potentially inefficient approach to expression imputation is used.

A small clarification: we did not only use the subjects that have data for all tissues, we used the subjects that have data for at least 60% of the tissues.

The sCCA methodology relies on the selection of tuning parameters that establish the level of sparsity in the genotype and expression data. There is a brief mentioning of using cross-validation for choosing c1 and c2 - but there are no details on how many folds should one select as a function of sample size and whether missing tissues have any impact on that choice. Note that cross-validation works poorly in regions with a lot of LD and when the number of subjects is small.

We have clarified that we use the *permutation* procedure implemented in the CCA.permute function in the PMA package to choose the tuning parameters c_1_ and c_2_, and we have added details about this procedure (lines 455-463). The CCA.permute procedure allows the user to specify how many permutations are used to choose the tuning parameters. The default is 25 permutations. We evaluated the sensitivity of our results to different numbers of permutations (from 5 to 25). The resulting sCCA features were consistent across these settings.

Correlation between sCCA features constructed using different values for nperms (N_permutaion) and the sCCA features constructed using nperms=25.

Given the similarity of these results, we set the number of permutations to 15 to reduce runtime.

An exhaustive evaluation of the best values for nperms or other approaches to selecting the tuning parameters across a wide range of plausible scenarios is beyond the scope of this paper.

A clarification is needed for the weight building step after sCCA: are the regressions run on all cis SNPs or only on the SNPs that have non-zero weights in the sCCA step? It seems from the paper that it is all SNPs - but it is not clear on why it is better to ignore the information (derived weights) from the sCCA step.

The regression is run on all the cis SNPs. Our algorithm consists of two steps: (1) build cross-tissue features; (2) build TWAS weights on the cross-tissue features. sCCA helps identify weighted averages of gene expression across tissues that are correlated with genotype. Given $\boldsymbol{\omega}_{\mathbf{2}}$**,** we use a the more flexible procedure implemented in FUSION to choose genotype weights to predict the cross-tissue feature $\mathbf{X}\boldsymbol{\omega}_{\mathbf{2}}$: because FUSION considers a broader range of prediction models (including ridge and elastic net besides LASSO), the weights from FUSION can improve prediction of the cross-tissue features. See also Reviewer 2’s first major comment and our response. We now note this on lines 478-484.

A couple of naive ways to do the multi-tissue analysis are: (i) average the expression across tissues (equal weights, partly justified by the cross-tissue findings for eQTls); (ii) weights that maximize the correlation with the top cis-eQTL (as opposed to using all SNPs). Does the sCCA approach work better than these naive approaches?

We did not directly compare these naïve approaches to the procedures we considered. But we believe approach (i) would have lower power relative sCCA. Even in the best case scenario for approach (i), where the absolute contribution of genetic variation to changes in gene expression is constant across all tissues, the relative contribution will vary, due to variation in the variability in expression levels across tissues. We also did not explicitly compare approach (ii), as it has been previously shown to be inefficient on average when constructing predictive expression-feature weights [3]. (Even when expression variation is driven by a single SNP, penalized regression approaches perform relatively well.)

On Table 1 - it is not clear if sCCA outperforms the competitors. I don't see it - but maybe I am reading the Table data incorrectly. The description on page 19 is not reflected in Table 1 - some clarification is needed.

Thanks for this comment. To avoid confusion, we have removed the “Total number of significant genes from TWAS Hub” column. Now it is clear to see that the number of significant genes by sCCA-TWAS and sCCA+ACAT approach significantly outperforms total number of significant genes in GTEx panel with single tissue TWAS approach. Including the “Total number of significant genes from TWAS Hub” column creates an apples-to-oranges comparison: “the total number of significant genes from TWAS Hub” also contains results using eQTL resources other than GTEx (e.g. large blood expression panels or TCGA). Moreover, the “total number of significant genes from TWAS Hub” is a simple union of the significant genes from each panel, not accounting for multiple testing from different panels. Our sCCA+ACAT results accounted for multiple gene expression panels used.

Minor comments:
(i) someone needs to look at the references; for example 9 (author?) and 16 (published in 2019).

We have fixed these two citations

(ii) middle of page 22 - P_1(omega_1) is not defined (is it the L1 norm or other sparsity inducing penalty?)

We have added the definition in the main text.

**Reviewer #2**

In this paper the authors discuss the problem of availability of the most relevant tissue in the context of transcriptome-wide association study. They propose a “TWAS using sparse canonical correlation analysis” approach, and demonstrate performance through simulation studies and real data application for various traits. Overall, this is an excellent manuscript. It is clearly written, logically built up and the method is solid. The simulation study is well-conducted and the real example is reasonable. There are a few issues I’d like to highlight in order to improve the manuscript.

Thanks for the kind words and the suggestions!

Major comments.
1. In sCCA-TWAS, the authors propose to use canonical variates Xu_1, Xu_2, and Xu_3 as the repeated measure of gene expression across tissue. Say Gv_1 is the corresponding canonical variate. The canonical correlation cor(Xu_1, Gv_1) is maximized, but it is not necessarily true that cor(Xu_1, G) is also maximized. How would the authors validate the choice of this particular linear combination of gene expressions across tissues instead of others?

Our original motivation was to leverage sCCA to construct cross-tissue features that capture any genetic contribution to genetic covariance (unlike the top components from PCA which need not capture the genetic contribution). As the reviewer notes, even if $\omega_{1}$ and $\omega_{2}$ maximize the correlation between $\mathbf{X}\boldsymbol{\omega}_{\mathbf{2}}$ and $\mathbf{G}\boldsymbol{\omega}_{\mathbf{1}}$ (subject to L_1_ constraints), there may be a different linear combination of **G** (chosen using a different set of constraints) that predicts $\mathbf{X}\boldsymbol{\omega}_{\mathbf{2}}$ with lower test-set error than $\mathbf{G}\boldsymbol{\omega}_{\mathbf{1}}$. This was our motivation for using elastic net to construct predictors of the cross-tissue expression features, as opposed to using $\boldsymbol{\omega}_{\mathbf{2}}$ directly.

Our primary goal was to propose an approach to constructing TWAS weights that improved TWAS power by leveraging pleiotropic effects on expression in multiple tissues. We do not claim that this approach is optimal. We note this in the discussion and stress that developing and evaluating alternative approaches to multi-tissue TWAS are important active areas of research (lines 412-423).

2. In the real data application, the authors report no novel discoveries or indications of previous results. For completeness, what biology has this new application revealed? Please discuss in the Discussion.

We report the significant genes identified by our approach in the Supplementary Table 5. Our primary focus is to propose a new analytic approach and benchmark its performance in simulations and real data. We are using “number of novel genetic associations identified” as a metric of performance in the real data. As with all genetic association studies, downstream *in silico* analyses and *in vitro* experiments work are certainly required to reveal new biology. This additional work requires more resources, more time and careful thought. We prefer not speculate on possible biologic mechanisms without this additional evidence, which should be the subject of future work. Releasing the list of significant results allows other groups to follow up on these results as well.

Minor comments.
Line 404. The authors might mean “tissues corresponding to the columns”? so that consistent with Line 459.

We have fixed the typo.

Line 409 & 415. Subscript missing in “omega”.

The missing subscripts have been added.

Line 418. What are X_1 and X_2? Not defined.

We have fixed the typo; these are now indicated as $\boldsymbol{G}^{\boldsymbol{T}}\boldsymbol{G}$ and $\boldsymbol{X}^{\boldsymbol{T}}\boldsymbol{X}$

Line 428. Are c1 and c2 chosen at each round or only for the first canonical variate pair?

c1 and c2 are chosen only for the first canonical variate pair. We have clarified this in the Methods (lines 455-463)

Line 478. What are p and q?

We have fixed this typo: p and q are now $p_{1}\mathrm{and}p_{2}$ (the number of columns in the **G** and **X** matrices, respectively).

Legends of the figure could be improved, so that it’s easier for readers to get the information.

We remade the figures and resubmitted them in higher resolution file format.

Reviewer #3: The authors proposed to use sparse canonical correlation analysis (sCCA) to construct cross-tissue features, and then combine both single-tissue TWAS and sCCA TWAS results by an aggregate Cauchy association test (ACAT) for TWAS. The authors showed that this sCCA-ACAT has the highest power in their simulation studies and identified the greatest number of significant genes in their real TWAS of 10 complex phenotypes. I have the following major comments:

1. I think the most comparable TWAS method the authors need to include in their comparison is the UTMOST instead of single tissue GBJ. Even though the UTMOST also used GBJ approach to aggregate test across multiple tissue types, the main feature of UTMOST is that it actually estimates TWAS weights jointly across multiple tissue types.

We have added UTMOST to the power comparison in Figure 3. The UTMOST results are similar single tissue GBJ; UTMOST has lower power than sCCA+ACAT and slightly better power than s-MultiXcan.

2. As shown in the simulation studies, using sCCA alone actually had the lowest power in the simulation studies, while combining sCCA and single tissue TWAS by ACAT gave the highest power. The authors might also be better to include Single Tissue ACAT into comparison to show that there will still be improvement for aggregating sCCA and single tissue TWAS, with the confounding factor of aggregating method ACAT excluded.

We note that sCCA did *not* have lowest power in all the situations we simulated. Consider the first panel in Figure 3, where the fraction of variability in causal-tissue expression values is low (*h*^2^=0.01): in that scenario, sCCA outperforms the methods based on combining single-tissue TWAS. This shows that sCCA gains power over the single-tissue-based approach when gene expression heritability is low, which is likely to happen in real data. The sCCA approach also outperforms the single-tissue based approach in the real data application as well. And the sCCA-ACAT approach gains yet more power by combining sCCA and single-tissue signals. (Conversely, when there is high heritability in the causal tissue, as in the second panel in Figure 3, single-tissue-based methods perform well.)

The increase in power for sCCA-ACAT relative to single-tissue-based methods is not simply due to differences between ACAT as a test-combining method and other methods used to combine single-tissue tests. Below is the power plot of ACAT applied to single-tissue results compared with GBJ, s-MultiXcan and Bonferroni in the scenario where half of the tissues are correlated with the causal tissue and causal tissue not included (i.e. the scenario presented in Fig. 3). ACAT has roughly equal power to these other methods to combine single-tissue results.

3. The authors mentioned Bonferroni correction for multiple tests in their TWAS comparison studies. Actually, it would worth to consider the number of independent test for Bonferroni correction, instead of taking 0.05/(number of tests) as the significance threshold. Otherwise, the Bonferroni correction will lead to conservative results, especially when the considered gene expression levels are correlated across multiple tissue types.

To clarify, the ACAT and GBJ tests do implicitly (ACAT) or explicitly (GBJ) account for test correlations. As the reviewer notes, Bonferroni correction may lead to loss of power when tests are correlated, and we’ve included it as a benchmark for this reason. We hypothesized that the ACAT and GBJ tests would outperform the Bonferroni correction---and they do.

4. As shown in S4 Fig, there is a huge inflation for testing significant heritability by sCCA method. I do not think showing the “power” for testing significant heritability in Fig 2 make any sense.

When developing expression feature weights for inclusion in TWAS, there is a trade-off between Type I and Type II errors at the expression-modeling stage. Stringent control of the Type I error when testing expression-feature heritability will increase Type II error and reduce the number of genes that can be tested at the TWAS stage. Figure S4 highlights that fact that the sCCA procedure identifies more features for testing at the TWAS testing stage, in part because it is implicitly making a different choice regarding the trade-off between Type I and Type II error at the expression-modeling stage. Importantly, we show that when the TWAS weights for the sCCA features are not associated with expression (Type I errors at the expression-modeling stage), Type I error is controlled at the TWAS testing stage, so the final TWAS associations retain appropriate control of false positives. We also show that including sCCA feature weights can improve power, even if some of the tested weights are not associated with expression. This suggests that on balance the additional genes that can be tested using sCCA feature weights include enough genes with truly genetically associated expression levels to more than counter balance the penalty from testing features that are not truly associated with genetic variation.

We have added this clarification to the discussion (lines 386-399).

5. For type I error evaluation of the TWAS method, the authors should consider using the genome-wide gene-based association study threshold 2.5e-6 as the significance level, instead of current 0.05.

We used 0.05 as the significance threshold to assess Type I error because it suffices to capture marked inflation in test statistics, and accurately estimating p-values on the order of 2.5e-6 is practically infeasible—it would require tens of millions of simulations for each scenario.

For power calculations, we used 0.05/2000=2.5e-5 to roughly account for the number of features actually tested in a typical TWAS (the number of genes with at least one heritable expression feature, as opposed to the number of genes across the genome that could possibly be tested). The relative performance of different tests is unchanged at the 0.05/20,000=2.5e-6 threshold (see following figure).

**Figure.** Power comparison for cross-tissue TWAS methods (as in Fig 3, except using 2.5e-6 as significance threshold rather then 2.5e-5).

6. Even though the sCCA/PCA approach provides some weights/coefficients for the gene expression of multiple tissue types, the sensitivity and specificity to identify the true relevant tissue type is around 0.5 and 0.7, with expression heritability 10% and correlation 0.3 with the causal tissue. This shows that it might still be challenging to identify the true causal tissue type?

Indeed. Our main aim was to increase TWAS power—the fact that this approach might yield any information at all that could be used to help identify the causal tissue is a bonus. We note that methods to identify the causal tissue are an important area for future research in the discussion (lines 414-415).

7. Besides providing a greater number of “significant” genes, the approach of testing the association between cis-SNPs and the sCCA/PCA features needs more justification in the perspective of biology. Does more mean higher power? What dose such a significant association mean?

Inferring underlying biology from any genetic association result is difficult and will require additional in silico and in vitro experiments. The biological motivation for our approach is the observed pleiotropy for expression in multiple tissues for some genes. Where the same cis variation influences expression values in multiple tissues, we aimed to take advantage of that. We show our approach increases power, leading to more candidates for future follow up. That follow up is beyond the scope of this paper. See also the response to Reviewer 2, major point 2.

8. Table 1 can be improved by using footnote to denote Reference papers and simplify text headers.

Thanks for this suggestion. We have removed the 4^th^ column of the table to make the table simpler and easier to read.

9. Figure 1 is hard to read. The authors may either split the flow chart per method, or only make one for the sCCA-ACAT method.

We remade the figure so that it only contains sCCA+ACAT approach.

10. The lines in Fig 3 are hard to see.

We have created higher resolution figures.

11. Figures 6, 7, 8 could be combined as 3 panels in one figure.

We have combined figure 6,7,8 into one figure.

12. All texts in the figures are hard to read.

Higher resolution figures have been provided as separate files upon resubmission.

References:

1. Slade, Emily Marie. 2018. Statistical Methods for Multi-Omics Data. Doctoral dissertation, Harvard University, Graduate School of Arts & Sciences.
2. Witten, D.M., Tibshirani, R. and Hastie, T., 2009. A penalized matrix decomposition, with applications to sparse principal components and canonical correlation analysis. *Biostatistics*, *10*(3), pp.515-534.
3. Gusev, A., Ko, A., Shi, H., Bhatia, G., Chung, W., Penninx, B.W., Jansen, R., De Geus, E.J., Boomsma, D.I., Wright, F.A. and Sullivan, P.F., 2016. Integrative approaches for large-scale transcriptome-wide association studies. *Nature genetics*, *48*(3), pp.245-252.
