# Supplementary material for: Leveraging expression from multiple tissues using sparse canonical correlation analysis and aggregate tests improves the power of transcriptome-wide association studies
Source: PLoS Genet. 2021 Apr 8;17(4):e1008973. doi: 10.1371/journal.pgen.1008973 (PMC8057593; doi:10.1371/journal.pgen.1008973)
Supplement: Attachment — Submitted filename: Response_To_Editors_05mar21.docx [file pgen.1008973.s015.docx]

We thank the editors for the opportunity to resubmit and their suggestion to apply UTMOST to the real-data application. Detailed responses to specific points follow.

While two of the reviewers were satisfied with the revision, one reviewer continued to express several concerns with the manuscript. After editorial discussions, we have decided to allow you an opportunity to submit a revised manuscript that must fulfill the following requirements:

1) Authors must confirm uploading of all sCCA cross-tissue feature weights into the TWAS HUB web interface

We confirm. Weights are available at: <http://gusevlab.org/projects/fusion/#reference-functional-data>. Note that this includes weights calculated using both version 6 of GTEx (used in this paper) and version 8.

2) Authors must provide a user manual and provide more detailed code documentation for their sCCA-TWAS software to enable the tool to be used by the broad genetics community

A user manual with documentation is available on github: <https://github.com/fenghelian/sCCA-ACAT_TWAS.>

Additionally, it is recommended that the authors apply UTMOST to their real data example in Table 1 and compare and contrast the number of significant genes identified by this existing method compared to the proposed methodology.

Done. See page 20, lines 364-374; pages 22-23, lines 427-433; and Supplementary Table 6.
